# Calenclar

**DOI:** 10.1155/S1463924681000461

**Published:** 1981


### Calendar

**Editor's Note:**
*Organisers of conferences, seminars etc. should send details for inclusion in this calendar as soon as the relevant information is available and not later than three months before the event.*

**Harmonisation of collaborative analytical studies**
August 20-21, Helsinki
*H Egan, Laboratory of the Government Chemist, Cornwall House, Stamford St, London SE1, UK*

**Euroanalysis IV**
August 23-28, Helsinki, Finland
*Euroanalysis IV, Association of Finnish Chemical Societies, Mr. Veikko Velamo, Executive Director, Pohj Hesperiankatu 3B 10, SF-00260 Helsinki 26, Finland*

**Analytical chemistry, 6th Australian symposium**
August 23-28, Canberra, Australia
*Honorary Secretary, Miss B.J. Stevenson, PO Box 1397, Canberra City, ACT 2601, Australia*

**XI International Congress of Clinical Chemistry/IV European Congress of Clinical Chemistry**
August 30 - September 5, Vienna
*Congress Secretariat, Interconvention, PO Box 35, A-1095, Austria*

**Expochem '81**
September 1-3, Houston
*Albert Zlatkis, Chemistry Department, University of Houston, Houston, Texas 77004, USA*

**Recent developments in instrumental chemical analysis**
September 7-9, Kingston-upon-Thames
*Mr. FA Burford, School of Chemical and Physical Sciences, Kingston Polytechnic, Penrhyn Rd, Kingston-upon-Thames, Surrey, UK*

**Laboratory '81 London**
September 8-10, London
*Curtis Steadman, 34-36 High Street, Saffron Waldon, Essex*

**Automated Analysis**
September 10-11, Stirling, UK
*Dr. C. J. Jackson, HSE, 403 Edgware Road, London NW2 6LN*

**Micro and Mini computers in the laboratory**
September 15-16, London
*Beverly Humphrey, 33-35 Bowling Green Lane, London, EC1R 0DA, UK*

**Filtech '81**
September 15-17, London
*Maureen Duck, Pressaids Ltd, Bridge House, 181 Queen Victoria St, London EC4*

**Computerised measurement**
September 22-25, Yugoslavia
*Jurema Secretariat, YU-41001 Zagreb, POB 398, Yugoslavia*

**Advances in chromatography - 16th International Symposium**
September 28 - October 1, Barcelona
*Albert Zlatkis, Chemistry Department, University of Houston, Houston, Texas 77004, USA*

**Application of microcomputers in measurement**
September 28-October 3, Yugoslavia
*Jurema Secretariat, YU-41001 Zagreb, POB 398, Yugoslavia*

**ILMAC '81: Laboratory, chemical engineering, measurement, and automation techniques in chemistry exhibition**
September 29-October 2, Basle
*Secretariat Ilmac '81, Postfach, CH-4021 Basle, Switzerland*

**2nd World Congress of Chemical Engineering**
October 4-9, Montreal
*Mr. Barry Bockus, Executive Secretary, Canadian Society for Chemical Engineering, 151 Slater St, Suite 906, Ottawa, Ontario, Canada K1P 5H3*

**Chemasia '81**
October 21-24, Singapore
*Andrew Dedman, Chemasia '81, Clapp & Poliak Europe Ltd, 232 Acton Lane, London W4 5DL*

**Petroanalysis '81**
October 27-29, London
*Irene McCann, Institute of Petroleum, 61 New Cavendish St, London W1, UK*

**Practical aspects of modern HPLC**

November 9-10, West Berlin

*1 Molnar, Wissenschaftliche Geratebau Dr Ing H Knauer GmbH, Hegauer Weg 38, D-1000, Berlin 37, GFR*

**Microprocessor-based equipment design and development**
November 9-13, Sevenoaks, Kent
*Conferences and Courses Unit, Sira Institute Ltd, South Hill, Kent BR7 5EH, UK*

**Viscosity measurement seminar**
November 10-11, London
Janet Bray, MSE Scientific Instruments, Manor Royal, Crawley, West Sussex.

**HPLC of proteins and peptides**
November 16-17, Washington DC, USA
*The Symposium Manager, International Symposium on HPLC of proteins and peptides, 400 East Randolph, Chicago, IL 60601, USA*

**1982**

**The Pittsburgh Conference and Exposition on Analytical Chemistry and Applied Spectroscopy**
March 8-12, Atlantic City
*Mr. Ralph M Raybeck, Exposition Chairman, Pittsburgh Conference, 437 Donald Rd, Pittsburgh, PA 15235, USA*

**12th Annual Symposium on the Analytical Chemistry of Pollutants**
April 14-16, Amsterdam.
*Prof Dr. R.W. Frei, Congress Office, 12th Annual Symposium on the Analytical Chemistry of Pollutants, Congress Bureau, Vrije Universiteit, PO Box 7161, 1007 MC Amsterdam, The Netherlands.*

**International Congress on Automation in the Clinical Laboratory**
April 19-22, Barcelona, Spain.
*Dr. R. Galimany, Departmento de Analisis Clinicos, Seccion de Automatizacion, Cuidad Sanitaria 'Principes de Espana', Hospitalet de Llobrogat, Barcelona, Spain.*

